# Accounting for density reduction and structural loss in standing dead trees: Implications for forest biomass and carbon stock estimates in the United States

**DOI:** 10.1186/1750-0680-6-14

**Published:** 2011-11-24

**Authors:** Grant M Domke, Christopher W Woodall, James E Smith

**Affiliations:** 1 USDA Forest Service, Northern Research Station, St. Paul, MN, USA; 2 USDA Forest Service, Northern Research Station, Durham, NH, USA

**Keywords:** carbon accounting, forest inventory, greenhouse gas, dead wood, snag, standing dead

## Abstract

**Background:**

Standing dead trees are one component of forest ecosystem dead wood carbon (C) pools, whose national stock is estimated by the U.S. as required by the United Nations Framework Convention on Climate Change. Historically, standing dead tree C has been estimated as a function of live tree growing stock volume in the U.S.'s National Greenhouse Gas Inventory. Initiated in 1998, the USDA Forest Service's Forest Inventory and Analysis program (responsible for compiling the Nation's forest C estimates) began consistent nationwide sampling of standing dead trees, which may now supplant previous purely model-based approaches to standing dead biomass and C stock estimation. A substantial hurdle to estimating standing dead tree biomass and C attributes is that traditional estimation procedures are based on merchantability paradigms that may not reflect density reductions or structural loss due to decomposition common in standing dead trees. The goal of this study was to incorporate standing dead tree adjustments into the current estimation procedures and assess how biomass and C stocks change at multiple spatial scales.

**Results:**

Accounting for decay and structural loss in standing dead trees significantly decreased tree- and plot-level C stock estimates (and subsequent C stocks) by decay class and tree component. At a regional scale, incorporating adjustment factors decreased standing dead quaking aspen biomass estimates by almost 50 percent in the Lake States and Douglas-fir estimates by more than 36 percent in the Pacific Northwest.

**Conclusions:**

Substantial overestimates of standing dead tree biomass and C stocks occur when one does not account for density reductions or structural loss. Forest inventory estimation procedures that are descended from merchantability standards may need to be revised toward a more holistic approach to determining standing dead tree biomass and C attributes (i.e., attributes of tree biomass outside of sawlog portions). Incorporating density reductions and structural loss adjustments reduces uncertainty associated with standing dead tree biomass and C while improving consistency with field methods and documentation.

## Background

The U.S. National Greenhouse Gas Inventory (NGHGI) produced annually by the U.S. Environmental Protection Agency recognizes five forest ecosystem carbon (C) pools [1]. Data from the USDA Forest Service, Forest Inventory and Analysis (FIA) program's network of permanent inventory plots across the Nation is used to either directly estimate (e.g., standing live trees) or simulate (e.g., litter) forest ecosystem C stocks. For example, C stock estimates for standing live tree biomass are based on inventory tree data, whereas estimates for down dead wood, litter, and soil organic matter are generated from models based on geographic area, forest type, and in some cases, stand age [2-4]. As the FIA inventory is the foundation for the U.S.'s NGHGI of managed forestland C stocks, improving the transparency and reliability of standing dead tree biomass and C stock estimation procedures is warranted. Currently, standing live and dead tree (SDT) biomass estimates are calculated using the same procedures in the FIA database [4]. It has been recognized that the density of dead wood generally decreases with each stage of biomass decay [5-8] and work is currently in progress to incorporate density reduction factors (DRF) into SDT biomass/C estimates in the FIA database [9]. There are also structural losses due to decomposition processes [10-13] which are not accounted for in the DRF. Sloughing and breakage resulting from biotic and abiotic activity over the course of decomposition should be considered in SDT biomass/C estimates to accurately account for biomass and C in forests. FIA qualitatively delineates five decay classes for SDTs based on decomposition characteristics for tree components (e.g., bark and crowns) (Table 1) [4,14]. Field crews are trained to adhere to classification descriptions when assigning SDTs to decay classes to ensure consistency [14]. Unfortunately, the descriptions are largely qualitative, and in some cases, are based on a single species in one region of the U.S. (e.g., Douglas-fir (*Psuedotsuga menziesii *(Mirb.) Franco)) [10]. While decay dynamics vary by site, species, and climatic region, the general trend in structural loss across these variables is likely similar throughout temperate and boreal forests [10-13,15]. Given the expected reduction in uncertainty and increased transparency in the U.S.'s NGHGI from incorporating and documenting decay and structural attributes of SDTs into their biomass/C estimation procedures, the objectives of this paper are to: 1) examine the distribution of SDTs across decay classes in the FIA database, 2) compare estimates of SDT biomass based on current and adjusted estimates (i.e., incorporation of decay reductions and structural loss deductions) by tree component, diameter, and decay class, 3) estimate differences in regional population estimates between current and adjusted biomass estimation procedures, and 4) suggest refinements of proposed SDT biomass/C estimation procedures and future research directions.

**Table 1:** Description of standing dead decay classes from USDA Forest Service [14].

Decay class	Description
1	Limbs and branches all present, top pointed, all bark remaining, sapwood intact, heartwood sound, hard, original color.

2	Few limbs and no fine branches present, top may be broken, bark variable, sapwood sloughing, heartwood sound at base incipient decay in outer edge of upper bole, hard, light to reddish brown.

3	Branches absent with only limb stubs, top broken, bark variable, sapwood sloughing, heartwood with incipient decay at base, advanced decay throughout upper bole, fibrous to cubical, soft, dark, reddish brown.

4	Branches absent with few or no stubs, top broken, bark variable, sapwood sloughing, heartwood with advanced decay at base, sloughing from upper bole, fibrous to cubical, soft, dark, reddish brown.

5	No limbs or branches, top broken, bark less than 20 percent, sapwood gone, heartwood sloughing, cubical, soft, dark brown, or fibrous, very soft, dark reddish brown, encased in hardened shell.

## Methods

Current methods for estimating SDT biomass and C stocks in the national FIA database are documented in Woudenberg et al. [4]. Tree level estimates of biomass and C are presently calculated identically for both live and SDTs as reflected in the tree table of the FIA database. This section provides an overview of DRF and structural loss adjustments (SLA) and describes the study areas and analysis. A detailed description of the volume-biomass-C conversion process along with biomass equations and example calculations may be found in Additional file 1.

### Density reduction factors

Currently, the density of live and SDTs in the FIA database is the same [4]; that is, there are no specific considerations for decay-related loss of organic material within the wood or other tree components. This may be the case in extremely dry environments where decomposition is slow. However, in most temperate and boreal environments, dead wood density is less than live tree density and decreases with increasing decay class [7-9]. To account for density reduction in dead wood, Harmon et al. [9] developed DRF for SDTs based on relationships between downed dead and SDT wood density as ascertained through field measurements across the northern hemisphere. Specifically, dead wood samples were categorized by decay class and divided into subsections where wood disks were cut (a cross section sample 5 to 10 cm thick) from each end and volume and weight measurements (wet and dry) were taken to determine the density of wood and bark [9]. DRFs were calculated as the ratio of the average current decayed density (current mass/volume) of the piece of dead wood to average undecayed (live tree mass/volume) density for each species and decay class (Table 2). DRFs were incorporated into current biomass and C estimation procedures for SDTs in this study to compare current biomass and C stock estimates with those adjusted for decay. Details on how DRF were incorporated into SDT biomass/C estimates may be found in Additional file 1.

**Table 2:** Density reduction factors by species [9] and preliminary SLA for each decay class by tree component for all tree species in the FIADB.

Decay class	Density reduction factors		Structural loss adjustment factors				
		
	Quaking aspen	Douglas-fir	Top	Bark	Bole	Stump	Roots
1	0.970	0.892	1.00	0.92	1.00	1.00	1.00
2	0.750	0.831	0.50	0.66	1.00	1.00	0.95
3	0.540	0.591	0.20	0.39	1.00	1.00	0.80
4	0.613	0.433	0.10	0.21	1.00	1.00	0.65
5	0.613	0.433	0.00	0.00	1.00	1.00	0.50

Note: values represent the proportion of original (live-tree equivalent) component biomass retained at each decay class.

### Structural loss adjustments

Structural loss or fragmentation in SDTs is widely documented in qualitative decay class descriptions [[10,14,16,17], and many others] and in studies of SDT longevity [5]; however, there are few quantitative references by decay class [13]. To remain consistent with the decay class descriptions in the FIA field guide [14], preliminary SLA were developed for SDT biomass components by decay class (Table 2). The preliminary SLA for top and branches and belowground biomass were estimated using qualitative descriptions from the FIA field guide [14] and other studies documenting structural loss by decay class and tree component [5,10,16,17]. Preliminary SLA for bark biomass were estimated from data collected as part of Harmon et al.'s [9] study. Merchantable stem deductions due to rough, rotten, or missing cull were accounted for in the conversion from gross to sound volume [4] so no additional SLA were estimated for bole or stump components (Table 2). SLAs were incorporated into current biomass and C estimation procedures for SDTs in this study to compare current biomass and C stock estimates with those adjusted for structural loss. Details on how SLA were incorporated into SDT biomass estimates may be found in Additional file 1.

### Component ratio method for calculating standing dead tree biomass

The component ratio method (CRM) was developed, in part, to facilitate estimation of tree component biomass from the central stem volume in standing live and SDTs [18]. SDTs in the FIA database are designated by a status code 2 and have a tree class code (general tree quality) 3 designating rough cull or 4 designating rotten cull [4]. Volume equations vary by region but generally tree class code 3 indicates that the tree is salvable (sound), while tree class code 4 indicates that the tree is nonsalvable (not sound). Gross to sound volume deductions are applied to all live and SDTs. The deductions are applied to the central stem and are carried forth to other tree components when converting sound volume to oven-dry biomass via the CRM [18,19]. A full description of the CRM along with equations and calculations may be found in Additional file 1.

### Regional case study

The most abundant SDT species in the Lake States (Michigan, Minnesota, and Wisconsin) and Pacific Northwest (Oregon and Washington) were selected to compare current biomass and C stock estimates with estimates which incorporate DRF and SLA. While the two species selected may not be representative of all species in their respective regions, they are both extremely common and provide a sound starting point for consideration. Quaking aspen (*Populus tremuloides *Michx.) is a short-lived, early successional hardwood species with a transcontinental range in North America [20]. It is the most common SDT species in the national FIA database and accounts for more than 18 percent of the SDTs in the Lake States region. Douglas-fir is a long-lived, moderately shade tolerant softwood species found throughout western North America [21]. It is one of the five most common SDT species in the FIA database and the most abundant SDT species in the Pacific Northwest.

**Figure 1:**
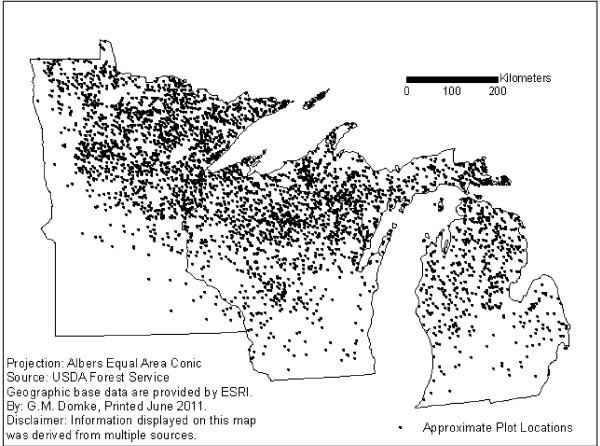
**Approximate plot locations of forest inventory plots with standing dead quaking aspen trees in the Lake States, 2005-2009**.

**Figure 2:**
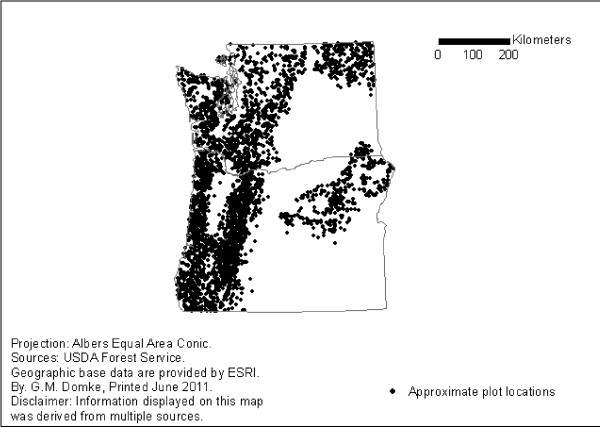
**Approximate plot locations of forest inventory plots with standing dead Douglas-fir trees in the Pacific Northwest, 2001-2009**.

Field data for each region and species were taken entirely from the FIA database. All SD aspen and Douglas-fir trees with a diameter at breast height (dbh) ≥ 12.7 cm were included in the analysis. A total of 9,369 SD aspen trees were sampled on 3,975 plots in the Lake States from 2005-2009 (Figure 1), and 10,144 SD Douglas-fir trees were sampled on 2,825 plots in the Pacific Northwest from 2001-2009 (Figure 2). Mean differences between SDT biomass estimates calculated using the CRM, CRM+DRF, and CRM+DRF+SLA were compared at the tree-level by tree component and decay class for the two species and regions using paired t-tests. Population estimates for each species and region were also evaluated to compare large-scale changes resulting from alternative biomass estimation procedures. Population estimates are based on the sum of the product of the known total area, the stratum weight, and the mean difference in standing dead biomass at the plot level for each species and stratum level [22]. The stratification approach is used to reduce the variance of attributes by portioning the population into homogeneous strata. To avoid the influence of stratification on the analysis, plot-level differences were assessed prior to stratification.

## Results

**Figure 3:**
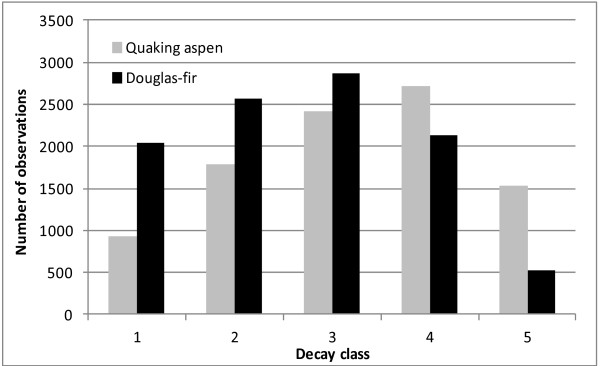
**Distribution of standing dead quaking aspen trees in the Lake States (2005-2009) and Douglas-fir trees in the Pacific Northwest (2001-2009) by decay class**.

The distributions of SD aspen and Douglas-fir trees tended toward a normal distribution centered around the third decay class (Figure 3). Nearly 29 percent of SD aspen were missing branches and an additional 16 percent lacked top and branch biomass. A detailed evaluation of the aspen decay class distribution by diameter class determined that small diameter stems (< 18 cm dbh) accounted for nearly 40 percent of the sample and were normally distributed across decay classes. More than 71 percent of aspen stems in each larger diameter class were found in decay classes 3, 4, and 5. For Douglas-fir stems in the Pacific Northwest, only 5 percent of sample trees had missing tops and branches and more than 73 percent of stems had at least some top, branch, and bark biomass present. More than 43 percent of the Douglas-fir trees sampled were less than 25 cm dbh, and of those, nearly 65 percent of the stems were in decay classes 1 and 2. Almost 69 percent of Douglas-fir trees greater than 25 cm dbh were in the advanced stages of decay, in classes 3, 4, and 5.

**Figure 4:**
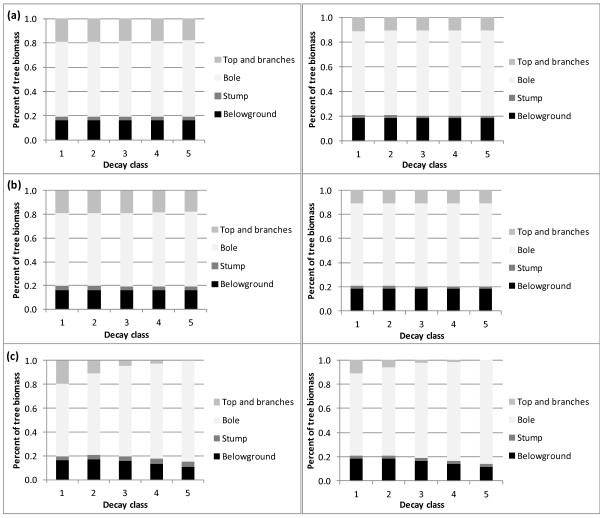
**Component ratios of tree-level oven-dry biomass by decay class and estimation method: a) CRM, b) CRM+DRF, and c) CRM+DRF+SLA for quaking aspen (left) in the Lake States (2005-2009) and Douglas-fir (right) in the Pacific Northwest (2001-2009)**.

**Figure 5:**
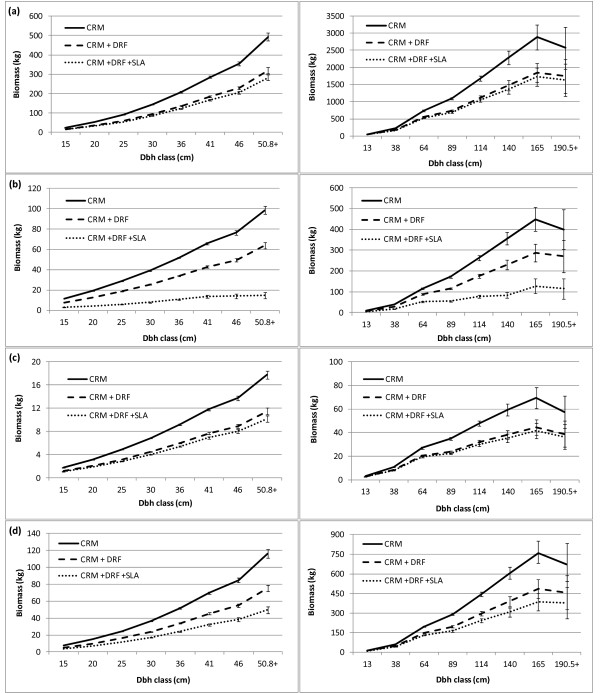
**Mean (with standard errors) standing dead oven-dry biomass (kg) by species (quaking aspen on left, Douglas-fir on right), estimation method, and dbh class for: a) bole, b) top and branches, c) stump, and d) belowground tree components**.

The distribution of biomass in individual SDTs was compared by decay class for the three biomass estimation approaches. The total biomass decreased with each adjustment by decay class, however the proportion of biomass in each tree component remained the same within the CRM and CRM+DRF (Figures 4 and 5). The proportion of bole biomass in the CRM and CRM+DRF increased slightly with increasing decay class, which resulted in a concomitant decrease in the biomass of other tree components. The distribution of biomass in the CRM+DRF+SLA changed substantially with increasing decay class (Figure 4). The proportion of top and branch biomass decreased from 19 percent in decay class 1 to 0 percent in decay class 5 for SD aspen and from 11 percent to 0 percent for SD Douglas-fir. Belowground biomass also decreased substantially by decay class in the two species and the combined deductions resulted in a proportional increase in bole biomass.

Mean differences in individual tree component biomass were compared across decay classes for the three estimation procedures. Incorporating DRF and DRF+SLA into the CRM for SDTs significantly decreased biomass estimates for all components and decay classes (Table 3). The largest decreases for both species occurred in the bole component of trees in advanced stages of decay. These differences are being driven by tree size and DRF, as no SLA are applied to the bole component.

**Table 3:** Mean tree-level differences in standing dead biomass (oven-dry kg) between estimation methods (1 = CRM vs. CRM+DRF and 2 = CRM vs. CRM + DRF+SLA) by tree component and decay class for quaking aspen in the Lake States (2005-2009) and Douglas-fir in the Pacific Northwest (2001-2009).

						Decay class					
		Quaking aspen						Douglas-fir			
			
Component	Comparison	1	2	3	4	5	1	2	3	4	5
Top and branches	1	0.8	6.7	11.8	8.7	8.4	9.7	16.7	37.7	4.0	33.8
	2	1.2	17.2	23.2	21.4	21.8	10.6	59.9	82.2	9.7	59.6
Bole	1	2.6	21.5	39.2	30.0	29.4	60.1	104.7	237.9	13.6	215.7
	2	3.8	25.2	44.0	36.5	37.3	65.8	130.0	268.2	16.5	239.6
Stump	1	0.1	1.1	2.0	1.5	1.5	2.2	3.6	7.6	0.7	6.4
	2	0.2	1.3	2.3	1.8	1.8	2.4	4.5	8.6	0.8	7.1
Belowground	1	0.7	5.7	10.3	7.8	7.6	16.2	28.0	63.4	3.5	57.1
	2	1.0	7.5	13.7	13.2	14.7	17.7	41.4	88.1	6.0	82.1

All means were significantly different at α = 0.05.

The disparity in individual tree biomass estimates was also evident by diameter class, in most cases, increasing with increasing diameter (Figure 5). Bole and stump biomass estimates were quite similar for the CRM+DRF and CRM+DRF+SLA for both study species across diameter classes, but substantially less than the CRM estimates. The CRM+DRF+SLA produced an almost uniform trend for top and branch biomass across diameter classes, while belowground biomass trends increased more or less consistently with the other two methods.

Differences in individual tree biomass for the three estimation procedures were also evident at the plot level across the two regions. The CRM+DRF and CRM+DRF+SLA significantly decreased plot-level SD bole biomass estimates for aspen by 65.8 and 78.1 kg, respectively across the Lake States (Table 4). In the Pacific Northwest, the CRM+DRF reduced plot-level SD Douglas-fir bole biomass by 595.0 kg and the CRM+DRF+SLA reduced bole biomass by 672.7 kg (Table 4).

**Table 4:** Mean plot-level difference (*d*) in standing dead biomass (oven-dry kg) by tree component and estimation method (1 = CRM vs. CRM+DRF and 2 = CRM vs. CRM+DRF+SLA) for quaking aspen in the Lake States (2005-2009) and Douglas-fir in the Pacific Northwest (2001-2009).

		Quaking aspen				Douglas-fir			
			
Component	Comparison	*t*	*df*	*p*	*d*	*t*	*df*	*p*	*d*
Top and branches	1	56.4	3966	< 0.001	19.6	21.4	2823	< 0.001	94.1
	2	56.8	3966	< 0.001	45.1	21.2	2823	< 0.001	203.1
Bole	1	48.1	3966	< 0.001	65.8	21.2	2823	< 0.001	595.0
	2	48.2	3966	< 0.001	78.1	21.2	2823	< 0.001	672.7
Stump	1	55.0	3966	< 0.001	3.4	23.4	2823	< 0.001	19.0
	2	55.2	3966	< 0.001	4.0	23.5	2823	< 0.001	21.5
Belowground	1	50.6	3966	< 0.001	17.2	21.3	2823	< 0.001	158.4
	2	49.9	3966	< 0.001	26.6	21.2	2823	< 0.001	216.5

**Figure 6:**
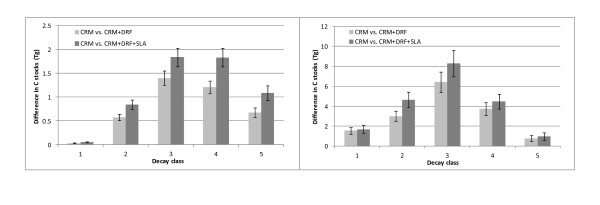
**Regional differences (with 95% confidence intervals) in C stocks (Tg) by estimation method (CRM vs. CRM+DRF and CRM vs. CRM+DRF+SLA) and decay class for quaking aspen (left) in the Lake States (2005-2009) and Douglas-fir (right) in the Pacific Northwest (2001-2009)**.

At a regional scale, CRM+DRF and CRM+DRF+SLA decreased total SD C stock estimates for aspen by 34 and 49 percent, respectively across the Lake States (Figure 6). In the Pacific Northwest, the CRM+DRF reduced regional SD Douglas-fir C stocks by almost 28 percent and the CRM+DRF+SLA reduced total C stocks by more than 36 percent (Figure 6). The largest regional changes were in the top and branch biomass for each species. Incorporating DRF into biomass estimation decreased aspen top and branch biomass by more than 34 percent and adding SLA into regional estimates reduced component biomass by nearly 78 percent. The inclusion of DRF in Douglas-fir top and branch biomass decreased regional SDT estimates by almost 24 percent and, combined with SLA, reduced estimates by nearly 60 percent.

## Discussion

Accounting for density reduction and structural loss in SDTs results in substantial changes to biomass and thus, C stock estimates, at multiple spatial scales. At the individual tree level, DRF correct for changes in SDT wood and bark specific gravity at each decay class. These corrections adjust biomass estimates for all SDT components relative to the current CRM, but because they are distributed to all SDT components in the estimation process, the distribution of biomass in each tree component remains the same. In some cases, dead wood density can increase as a result of shell hardening [23]. This commonly occurs in advanced stages of decay where outer wood tissues dry out, creating a hardened shell around inner tissue which continues to decompose [24]. Shell hardening has been documented in several hard- and softwood genera which have been standing for long periods of time [23,24]. There is evidence of shell hardening in both species in this study, which is reflected by increases in the DRF between decay classes 3 and 4. Despite these increases, mean biomass estimates for individual SDTs decrease with increasing decay class. This is due to successively larger volume deductions made for rough, rotten or missing cull in the merchantable stem prior to biomass conversion in each decay class.

Incorporating SLA into individual tree biomass calculations further reduces tree component biomass estimates. Structural loss is well documented in the decay class description in FIA's inventory documentation as well as studies using similar classification systems. Despite consistent decay class descriptions documenting structural loss in tree components, there is a dearth of empirical information available to develop SLA. The preliminary SLA presented in this paper are based on decay class descriptions and, where available, preliminary data (e.g., bark biomass; [9]) were used to develop adjustments. In the case of top and branch biomass, most decay class descriptions state that limbs and branches are absent in class 4 and tops are absent in class 5. In these cases, the descriptions were interpreted literally with no top and branch biomass, resulting in significant differences for this component across estimation methods. No structural loss was assumed for merchantable stem biomass since adjustments were made for rough, rotten, and missing cull prior to biomass conversion [4]. A SLA was included in bark biomass estimates so bole biomass, which includes merchantable stem and bark biomass, was adjusted for density reductions and structural loss.

Not surprisingly, differences in regional biomass estimates for the two species in this study were consistent with individual tree and plot-level trends for the different estimation procedures. The significant reduction in SDT biomass highlights the importance of including decomposition dynamics in forest ecosystem biomass and C stock estimates. The absolute differences for each region and estimation method likely reflect the largest differences expected nationally, given the species selected were the most abundant live and SDTs in their respective regions. That said, the proportion of live to SDTs for each species in this study was consistent with the mean live to SDT ratios for all species in each region which lends confidence in the results. While the focus here was on the two example species, the results summarized for the two reflect broad general differences between East and West in terms of SDTs as currently represented in the annual inventory. In the East, a greater proportion of forested plots are likely to include SDTs and assigned decay class codes are likely to be higher, relative to forested plots in the West.

The preliminary DRF and SLA for SDTs presented in this study are based on a relatively small number of species studied in a few regions across the northern hemisphere. While the general trends provide a starting point for SDT adjustments, species-specific data on density reductions and structural loss by tree component and decay class are necessary to further refine SDT biomass and C stock estimates. Existing information in the FIA database, such as core optional variables like actual tree height and total tree height, may be used to estimate broken or missing top biomass in SDTs. This represents a potential starting point for empirically based SLA, however there must also be a priority to improve linkages between field protocol descriptions of SDT decay classes and component estimation procedures. For example, the CRM for SDT top and branch biomass does not adhere to the descriptions for decay classes 4 or 5 in the FIA field guide. Furthermore, there are currently no qualitative SDT decay class descriptions for decomposition dynamics in coarse roots. In some species and regions, this may be appropriate; in other cases, however, it may not be. Defining structural loss by tree component and decay class for all SDTs may be one approach within the current inventory system. This would require additional training for field crews, increase the time spent on each plot, and raise sampling costs. However, it would maintain current estimation procedures with the adjustments described herein. Alternatively, a new method for estimating tree volume, biomass, and C stocks may be needed which is not based on merchantability standards and fully incorporates procedures necessary for SDTs. Such a method would likely require new field protocols to account for rough, rotten, and missing volume in each live and SDT component and decay class, resulting in additional costs for training and personnel. The costs of developing a new estimation procedure would have to be weighed against the potential benefits, be they improvements in accuracy, consistency, and efficiency of generating biomass and C stock estimates.

## Conclusions

National scale forest resource inventories in the U.S. have evolved from a timber-centric focus toward a more inclusive sampling of forest ecosystem attributes such as C stocks of standing dead trees. Likewise, the estimation procedures associated with such a forest inventory evolution need to be inclusive of tree attributes beyond those required by the forest products industry (e.g., board foot volumes of growing stock live trees). Developing SDT biomass and C stock estimates within the construct of an inventory system traditionally designed to estimate growing stock volume requires: 1) the development of a SDT decay class system which is both qualitative for ease of use in the field and quantitative to account for structural loss by tree component and species, 2) the development of DRF for SDT species in each decay class, with specific emphasis on advanced decay classes, and 3) the development of a flexible SDT estimation procedure which incorporates initial structural loss and density reduction information and allows for continual refinement.

SDTs are an important part of the dead wood forest ecosystem C pool recognized by the international community. In an effort to improve the accuracy and consistency of biomass and C stock estimates that are used in various facets of the U.S.'s national forest inventory, preliminary DRF and SLA have been developed for SDTs. These adjustments reflect the current state of the science on SDT biomass/C estimation and result in significant decreases in individual tree- and plot-level biomass estimates, and thus, substantial decreases in regional SDT biomass and C stock estimates. The results from this study suggest that incorporation of the SDT adjustments will significantly reduce estimates of dead wood biomass and C stocks across spatial-scales and forest types of the U.S. While the preliminary values offer a sound starting point for SDT biomass/C estimation, more work is necessary to refine SLA, perhaps by species and region, for each decay class used in national inventory field sampling.

## List of abbreviations

C: Carbon; CRM: Component ratio method; DRF: Density reduction factors; FIA: Forest Inventory and Analysis; FIADB: Forest Inventory and Analysis Database; NGHGI: National Greenhouse Gas Inventory; SDT(s): Standing dead tree(s); SLA: Structural loss adjustments.

## Competing interests

The authors declare that they have no competing interests.

## Authors' contributions

GMD performed the analysis and was a contributing author. CWW provided advice throughout the analysis and was a contributing author. JES provided advice throughout the analysis and was a contributing author. All authors have read and approved the final manuscript.

## Supplementary Material

Additional file 1 **Standing dead tree biomass equations and example calculations**. This file presents equations necessary to estimate above and belowground SDT biomass and C stocks and provides example calculations for reference [25,26]. Click here for file
