# Supplementary material for: Accounting for density reduction and structural loss in standing dead trees: Implications for forest biomass and carbon stock estimates in the United States
Source: Carbon Balance Manag. 2011 Nov 24;6:14. doi: 10.1186/1750-0680-6-14 (PMC3283479; doi:10.1186/1750-0680-6-14)
Supplement: Additional file 1 — Standing dead tree biomass equations and example calculations. This file presents equations necessary to estimate above and belowground SDT biomass and C stocks and provides example calculations for reference [25,26]. [file 1750-0680-6-14-S1.PDF]

Additional files

Additional file 1 - Standing dead tree biomass equations and example calculations

This file presents equations necessary to estimate above and belowground SDT biomass and C stocks and provides example calculations for reference.

Standing dead biomass equations

A. Volume to merchantable bole biomass

Gross volume (VOLCFGRS) is calculated using regional equations which rely on height (or height surrogates such as site index) and dbh values to estimate total volume in the central stem of the tree [19]. Sound volume (VOLCFSND) for live and SDTs is calculated from VOLCFGRS by multiplying deductions for rough, rotten, or missing volume in the central stem. In most regions within the contiguous U.S., gross to sound volume deductions are based on the estimated percent of cubic-foot volume that is rotten or missing. In the North Central region (IA, IL, IN, KS, MI, MO, MN, ND, NE, SD, WI), sound volume equations incorporate standard volume deduction coefficients for each TREECLCD [19].

For a complete description and list of current FIA volume equations and deductions, see Woodall et al. [19]. Sound volume estimates are multiplied by wood specific gravity and the density of water to convert to merchantable stem biomass. The same steps are used to calculate bark biomass only replacing wood specific gravity with bark specific gravity. The sum of bark and merchantable stem biomass is the merchantable bole biomass.

B. Proposed SDT decay and bark structural reductions: merchantable bole biomass

Merchantable bole biomass is used in conjunction with biomass estimates from Jenkins et al. [25] to estimate tree component biomass via an adjustment factor in the CRM [19]. Since the effects of the DRF [9] and bark SLA (Table 2) are intended for all SDT components, they are applied to the merchantable stem and bark biomass prior to calculating the adjustment factor (step C), which is subsequently applied to the component estimates. Both deductions are based on the decay class assigned to each sample tree.

Stem wood biomass:

$$B_{odw} = V_{gw} * SG_{gw} * W * DRF * BOLE\_SLA \qquad \text{(eq. 1)}$$

where

B_{odw} = oven-dry biomass (kg) of wood

V_{gw} = sound volume of green wood in the central stem

SG_{gw} = basic specific gravity of wood (oven-dry mass of green volume) from REF_SPECIES file in Woodall et al. [19]

W = weight m^3 of water (1000 kg)

DRF = density reduction factor

$Bole\_SLA$ = bole SLA (Table 2)

Bark biomass:

$$B_{odb} = V_{gw} * BV\% * SG_{gb} * W * DRF * BARK\_SLA \qquad \text{(eq. 2)}$$

where

B_{odb} = oven-dry biomass (kg) of bark

V_{gw} = sound volume of green wood in the central stem

BV% = bark as a percentage of wood volume from REF_SPECIES file in Woodall et al. [19]

SG_{gb} = basic specific gravity of bark (oven-dry mass of green volume) from REF_SPECIES file in Woodall et al. [19]

W = weight m^3 of water (1000 kg)

DRF = density reduction factor (Table 2)

BARK_SLA = bark SLA (Table 2)

Total SDT bole (bole and bark) biomass:

$$B_{odt} = B_{odw} + B_{odb} \quad \text{(eq. 3)}$$

where

B_{odt} = total SDT oven-dry bole biomass (kg)

B_{odw} = oven-dry biomass (kg) of stem wood

B_{odb} = oven-dry biomass (kg) of bark

C. Merchantable bole biomass to tree component biomass

Once merchantable bole biomass and the corresponding Jenkins biomass have been calculated for the sample tree, a CRM adjustment factor can be calculated by dividing standing dead bole biomass by Jenkins bole biomass. This factor is what carries the DRF and bark SLA forth to other tree components. The CRM adjustment factor is multiplied by each Jenkins component biomass estimate to estimate tree component biomass values adjusted for the CRM, which are proportioned according to the regional volume equations.

Standing dead CRM adjustment factor:

$$CRM_{AdjFac} = \frac{B_{odt}}{MST} \qquad \text{(eq. 4)}$$

where

CRM_{AdjFac} = component ratio method adjustment factor for SDT components

B_{odt} = total SDT oven-dry bole biomass (kg) (eq. 3)

MST = merchantable oven-dry bole biomass (kg) from Jenkins et al. [25]

D. Proposed SDT structural reductions: tree component biomass

Tree component estimates are multiplied by the SLA (Table 2) according to the assigned decay class for the sample tree.

Stump volume:

$$S_{vosb\ or\ visb} = \left(\frac{\pi(dbh)^2}{4(144)}\left[\left((A-B)^2 h + 11B(A-B)\ln(h+1) - \frac{30.25}{h+1}B^2\right)\right]_a^b\right)0.02832 \qquad \text{(eq. 5)}$$

where

$S_{vosb\ or\ visb}$ = stump volume inside bark (*visb*) or outside bark (*vosb*) (m^3)

A = Coefficient (species parameter) from Table 2 in Raile [26]

B = Coefficient (species parameter) from Tables 1 and 2 in Raile [26]

h = height aboveground (ft)

ln = natural logarithm

a = lower stump height (ft) – 0 ft in FIADB

b = upper stump height (ft) – 1 ft in FIADB

Stump wood biomass:

$$S_{odsw} = S_{visb} * SG_{gw} * W \qquad \text{(eq. 6)}$$

where

S_{odsw} = oven-dry biomass (kg) of stump wood

S_{visb} = stump volume inside the bark (eq. 5)

SG_{gb} = basic specific gravity of wood (oven-dry mass of green volume) from REF_SPECIES file in Woodall et al. [19]

W = weight m^3 of water (1000 kg)

Stump bark biomass:

$$S_{odsb} = (S_{vosb} - S_{visb})SG_{gb} * W \qquad \text{(eq. 7)}$$

where

S_{odsb} = oven-dry biomass (kg) of stump bark

S_{vosb} = volume (m^3) of stump outside the bark (eq. 5)

S_{visb} = volume (m^3) of stump inside the bark (eq. 5)

SG_{gb} =basic specific gravity of bark (oven-dry mass of green volume) from REF_SPECIES file in Woodall et al. [19]

W = weight m^3 of water (1000 kg)

Total SDT stump (wood and bark) biomass:

$$S_{odt} = (S_{odsw} + S_{odsb})CRM_{AdjFac} * STUMP\_SLA \qquad \text{(eq. 8)}$$

where

S_{odt} = SDT oven-dry stump biomass (kg)

B_{odw} = oven-dry biomass (kg) of wood (eq. 6)

B_{odb} = oven-dry biomass (kg) of bark (eq. 7)

CRM_{AdjFac} = component ratio method adjustment factor (eq. 4)

STUMP_SLA = stump SLA (Table 2)

Standing dead top and branch biomass:

$$T_{odt} = (TAB - MST - STP - FOL)CRM_{AdjFac} * Top\_SLA \quad \text{(eq. 9)}$$

where

T_{odt} = oven-dry biomass (kg) of standing dead top and branches

TAB = total aboveground oven-dry biomass (kg) [25]

MST = merchantable stem oven-dry biomass (kg) [25]

STP = stump oven-dry biomass (kg) [26]

FOL = foliage oven-dry biomass (kg) [25]

CRM_{AdjFac} = component ratio method adjustment factor (eq. 4)

Top_SLA = top and branch SLA (Table 2)

Standing dead belowground biomass:

$$BG_{odt} = ROOT * CRM_{AdjFac} * BG\_SLA \quad \text{(eq. 10)}$$

where

BG_{odt} = oven-dry biomass (kg) of standing dead coarse roots (≥ 0.254 cm diameter)

CRM_{AdjFac} = component ratio method adjustment factor (eq. 4)

BG_SLA = belowground SLA (Table 2)

E. Biomass to carbon

Component biomass estimates adjusted for density reductions and structural loss are multiplied by 0.5 to convert oven-dry biomass to carbon.

Standing dead tree example calculations

Below is an example of standing dead biomass calculations for a 26.7 cm quaking aspen tree in decay class 3 found in the Lake States region. Note that values may not be exact due to rounding.

A. Volume to merchantable bole biomass

1. Gross volume (VOLCFGRS) = 0.377 m^3
2. Sound volume (VOLCFSND) = 0.202 m^3

B. Proposed SDT decay and bark structural reductions: merchantable bole biomass

3. Stem wood biomass (eq. 1): $B_{odw} = V_{gw} * SG_{gw} * W * DRF * BOLE\_SLA$ = 0.202 * 0.35 * 1000 * 0.540 * 1 = 38.2 kg
4. Bole bark biomass (eq. 2): $B_{odb} = V_{gw} * BV\% * SG_{gb} * W * DRF * BARK\_SLA$ = 0.202 * 0.144 * 0.5 * 1000 * 0.540 * 0.39 = 3.1 kg
5. Total bole biomass (eq. 3): $B_{odt} = B_{odw} + B_{odb}$ = 38.2 + 3.1 = 41.3 kg

C. Merchantable bole biomass to tree component biomass

6. CRM adjustment factor (eq.4): $CRM_{AdjFac} = \frac{B_{odt}}{MST} = \frac{41.3}{201.8} = 0.2$

D. Proposed SDT structural reductions: tree component biomass

7. Stump volume (eq. 5): $S_{vosb} = \frac{\pi(dbh)^2}{4(144)}\left[\left((A-B)^2h + 11B(A-B)\ln(h+1) - \frac{30.25}{h+1}B^2\right)\right]_a^b =$

$\frac{\pi(10.5)^2}{4(144)}\left[\left((1-0.09658)^2 1 + 11 * 0.09658(1-0.09658)\ln(1+1) - \frac{30.25}{1+1}0.09658^2\right) - \left((1-0.09658)^2 0 + 11 * 0.09658(1-0.09658)\ln(0+1) - \frac{30.25}{0+1}0.09658^2\right)\right]0.02832 =$

0.0276 m^3

$S_{visb} = \frac{\pi(10.5)^2}{4(144)}\left[\left((0.91882-0.08593)^2 1 + 11 * 0.08593(0.91882-0.08593)\ln(1+1) - \frac{30.25}{1+1}0.08593^2\right) - \left((0.91882-0.08593)^2 0 + 11 * 0.08593(0.91882-0.08593)\ln(0+1) - \frac{30.25}{0+1}0.08593^2\right)\right]0.02832 = 0.0230\ m^3$

8. Stump wood biomass (eq. 6): $S_{odsw} = S_{visb} * SG_{gw} * W$ = 0.0230 * 0.35 * 1000 = 8.1 kg

9. Stump bark biomass (eq. 7): $S_{odsb} = (S_{vosb} - S_{visb})SG_{gb} * W$ = (0.0276 – 0.0230)0.5 * 1000 = 2.3 kg

10. Total stump biomass (eq. 8): $S_{odt} = (S_{odsw} + S_{odsb})CRM_{AdjFac} * STUMP\_SLA$ = (8.05 + 2.30)0.20 * 1 = 2.1 kg

11. Top and branch biomass (eq. 9): $T_{odt} = (TAB - MST - STP - FOL)CRM_{AdjFac} * Top\_SLA$ = (277.94 – 201.79 – 10.36 – 5.85)0.20 * 0.20 = 2.5 kg

12. Belowground biomass (eq. 10): $BG_{odt} = ROOT * CRM_{AdjFac} * BG\_SLA$ = 52.82 * 0.20 * 0.80 = 8.6 kg

E. Biomass to carbon

13. Total aboveground biomass = $B_{odt} + S_{odt} + T_{odt}$ = 41.3 + 2.1 + 2.5 = 45.9 kg

14. Total above and belowground biomass = 45.9 + 8.6 = 54.5 kg

15. Total aboveground carbon = $(B_{odt} + S_{odt} + T_{odt})0.5$ = (41.3 + 2.1 +2.5)0.5 = 23.0 kg C

16. Total above and belowground carbon = 54.5 * 0.5 = 27.3 kg C
